# The use of respondent-driven sampling to assess malaria knowledge, treatment-seeking behaviours and preventive practices among mobile and migrant populations in a setting of artemisinin resistance in Western Cambodia

**DOI:** 10.1186/s12936-017-2003-9

**Published:** 2017-09-19

**Authors:** Po Ly, Julie Thwing, Colleen McGinn, Cesia E. Quintero, Narann Top-Samphor, Najibullah Habib, Jack S. Richards, Sara E. Canavati, Seshu Babu Vinjamuri, Chea Nguon

**Affiliations:** 1 National Centre For Parasitology, Entomology and Malaria Control, Ministry of Health, Corner Street 92, Rapaing Svay Village, Sankat Phnom Penh Thmey, Khan Sensok, Phnom Penh, Cambodia; 2 Malaria Branch, Division of Parasitic Disease and Malaria, Centres for Disease Control and Prevention, Atlanta, USA; 3 WHO Representative Office in Cambodia, # 61-64 Norodam Preah Blvd., Penh, Phnom Penh, Cambodia; 4 Centre for Biomedical Research, Burnet Institute, Melbourne, Australia; 5 Vysnova Partners Inc., Washington, DC USA

**Keywords:** Respondent-driven sampling, Mobile and migrant populations, Western Cambodia, Containment project, Artemisinin resistance, Malaria elimination, Operational research, Malaria, KAP

## Abstract

**Background:**

Multi-drug-resistant *Plasmodium falciparum* threatens malaria elimination efforts in Cambodia and the Greater Mekong Subregion (GMS). Malaria burden in the GMS is higher among certain high-risk demographic groups in Cambodia, especially among migrant and mobile populations (MMPs). This respondent driven sampling (RDS) study was conducted in order to determine malaria knowledge, treatment-seeking behaviours and preventive practices among two MMP groups in Western Cambodia.

**Methods:**

An RDS survey of MMPs was implemented in four purposively-selected communes along the Thai–Cambodia border; two in Veal Veang District and two in Pailin Province, chosen due to their sizeable MMP groups, their convenience of access, and their proximity to Thailand, which allowed for comparison with RDS studies in Thailand.

**Results:**

There were 764 participants in Pailin Province and 737 in Veal Veang District. Health messages received in Veal Veang were most likely to come from billboards (76.5%) and family and friends (57.7%), while in Pailin they were most likely to come from sources like radio (57.1%) and television (31.3%). Knowledge of malaria transmission by mosquito and prevention by bed net was above 94% in both locations, but some misinformation regarding means of transmission and prevention methods existed, predominantly in Veal Veang. Ownership of treated bed nets was lower in Pailin than in Veal Veang (25.3% vs 53.2%), while reported use the night before the survey was higher in Pailin than in Veal Veang (57.1% vs 31.6%). Use of private sector health and pharmaceutical services was common, but 81.1% of patients treated for malaria in Pailin and 86.6% in Veal Veang had received a diagnostic test. Only 29.6% of patients treated in Pailin and 19.6% of those treated in Veal Veng reported receiving the indicated first-line treatment.

**Discussion:**

Barriers in access to malaria prevention and case management were common among MMPs, with marked variation by site. Resolving both nation-wide and MMP-specific challenges will require targeted interventions that take into account this heterogeneity.

## Background

Malaria control interventions have resulted in an 81% decrease in *Plasmodium falciparum* malaria cases in Cambodia over the last decade [1]. Nevertheless, the emergence and spread of *P. falciparum* resistance to artemisinin and partner drugs throughout Cambodia and the Greater Mekong Subregion (GMS) threatens these advances, and poses an alarming threat to global malaria mortality rates [2]. While resistance to multiple anti-malarial drugs has long plagued Western Cambodia, resistance to artemisinin was first found on the Cambodia–Thailand border in a series of studies conducted between 2007 and 2009. Resistance to artemisinin and partner combination drugs has been confirmed in multiple sites across the GMS [2–4], but Western Cambodia remains a particular hotspot [5, 6]. Like *P. falciparum* drug resistance, malaria transmission hot spots in the GMS are mostly located in the border regions of Cambodia, Thailand, Laos, Vietnam and Myanmar [7]. High rates of human population movement across those borders serve to transport malaria internationally, posing a threat to malaria elimination [8]. The same applies to intra-national migration, a process by which malaria can be reintroduced to regions that had previously achieved elimination [9]. To counter this, there have been reports of successful cross-border screening and treatment interventions [8]. The significant challenges posed by migration and spread of malaria cannot be effectively addressed without a thorough understanding of the populations that are most affected by malaria in the GMS.

In malaria elimination settings, decreasing transmission rates concentrate the burden of malaria among demographic groups who engage in certain high-risk behaviours [10–13]. In Western Cambodia, those most at risk include those who live in remote areas, as well as mobile and migrant populations[9]. The latter often live and work in areas with high malaria transmission and high human-vector contact, such as forests and forest-fringe areas [9, 14]. MMPs are often illiterate, impoverished, and poorly connected to public health and surveillance systems, including village malaria workers (VMWs), clinics, and reputable pharmacists. They are more likely to seek care from unregulated, private vendors, which may increase their risk of exposure to substandard and counterfeit drugs, or artemisinin monotherapy [15]. Their high mobility makes them difficult to reach with health promotion messages, and newcomers to endemic areas from non-endemic areas are at higher risk of contracting infections, because they have not been exposed to the educational and preventive interventions targeted to endemic regions [16]. These factors all combine to spread drug resistance and undermine malaria elimination efforts [8, 17, 18].

Mobile and migrant populations are often targeted by interventions as a homogenous group, but in actuality they have varying patterns of health behaviour and utilization of health services [19]. Work in Pailin Province is primarily agricultural, with defined work seasons for specific crops. Both male and female migrant workers typically live in communal dwellings, and they often stay for several seasons at a time. MMPs in Pailin most often fit the previously published MMP profile of *Seasonal Agricultural Workers*. In Veal Veang, agricultural workers tend to be more mobile and less settled than those in Pailin, falling into the *Periodic Agricultural Worker* category. There is also a migrant workforce, mostly male, involved in logging, which would we categorized as *Periodic Forest Workers*. They tend to have shorter stays, and live deep in the forest, sleeping under makeshift shelters [16].

While geographically stable populations can be located and studied easily with community-based surveys, mobile and relatively hidden populations, such as the farm and forest workers who are the focus of this study, are much more difficult to locate, enumerate, and follow up. Respondent-driven sampling (RDS) was developed as a method of achieving reliable, statistically robust estimates for difficult-to-access populations for whom sampling frames may be impossible to generate [20]. The RDS method is a modified form of snowball sampling, which allows researchers to recruit groups that do not congregate in stable and identifiable places [21].

This paper is the first publication that presents data from an RDS study on malaria knowledge, treatment-seeking behaviours and preventive practices in Cambodia. This insight can aid practitioners and policymakers in developing interventions that can better reach these high-risk groups.

## Methods

### Study area and population

The study was implemented in Pailin Province (pop. 70,482), a small border province at the northern edge of the Cardamom Mountains, and in the Veal Veang District (pop. 57,523) of the nearby Pursat Province. These sites were selected due to a high incidence of malaria, comparable overall population sizes, sizeable number of MMPs, and because both were regions known to have artemisinin resistance [22]. They were also selected due to their location on the Thai–Cambodia border, which would allow for comparison with similar RDS studies in Thailand.

Within each study site, two communes were selected to conduct the surveys: Andong 2 and Pang Rolem Communes in Pailin Province, and Chhay Louk and Pramuoy Communes in Veal Veang District, chosen for their ease of access to the often remote MMP groups, critical for the success of the RDS methodology. In addition, the field team ensured that the survey locations were in conspicuous places that were convenient, accessible, and of interest to both male and female potential respondents from the target populations. Each of the four survey sites had a staff team of two men and two women, who were contracted to work full-time at these sites for the duration of the study. All were trained in each of the key roles for the survey locations; site supervision, coupon and incentive management, screening and obtaining consent, conducting interviews and coordinating appointments.

### Study tools

The questionnaire used for the study was adapted by the technical team from a recent RDS study conducted in Thailand as part of the WHO’s Artemisinin-Resistance Containment Project [23, 24]. Some questions mirrored those used in the Thai study to allow for direct comparison of results between the two studies. The questionnaire was then translated into Khmer and included sections on sociodemographics, migratory patterns, work history, access to health messages, knowledge about malaria, malaria prevention activities including personal protective measures, treatment-seeking behaviour, treatment received and a section designed to measure the extent and depth of social networks.

### Sampling

Sample sizes were calculated separately for migrants in Pailin Province and Veal Veang District, in order to account for the non-overlapping social networks of the two study sites. Sample sizes were calculated using a conservative target proportion of 50% with a confidence level of 95% and a confidence interval of 0.45, 0.55. A design effect of 2.0 and a non-response rate of 10% were applied. A sample size of 675 participants for each of the two districts (total 1350 participants) was, therefore, considered sufficient.

### Recruitment

**Fig. 1:**
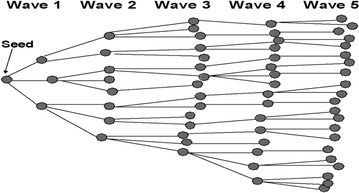
Seeds and recruitment chain

Following training, field-testing, and final revisions of the questionnaire, the initial seeds were selected (two men and two women in each of the four survey locations, total of 16). Seeds are the first participants, who are chosen non-randomly by the study staff at the beginning of the study. Local VMWs and other contacts assisted the field team in identifying the initial seeds, to ensure that they were representative of the MMPs at that study site, and that they were well-known and respected. Each seed was asked to recruit three other participants. The first group of recruited participants is referred to as the first wave. The first wave then recruits another three participants each (i.e. second wave) and so on, until the desired sample size is reached (Fig. 1). With each successive wave, the sample grows closer to equilibrium, meaning that sample characteristics reach a steady state that does not change with successive waves. Data collection was conducted between November 2010 and January 2011.

### Incentives

Each participant was given an incentive and an insecticide-treated net (ITN) after they had participated in the questionnaire. The team also reimbursed participants for reasonable travel expenses. The participant also received an incentive for each of up to three recruits after their recruit participated in the survey. Initially, each participant received three recruitment coupons to distribute to members of their social network; this was reduced to lower numbers of coupons as the sample size approached its target.

### Data management and oversight

The recruitment was controlled by a coupon management system, which was developed in Microsoft Office Excel version 2007. This system was used to track the relationships between the recruiters and their recruits. The questionnaires were administered on paper and data were double-entered. Data quality on the survey questionnaires was checked and reconciled with the coupon management system. Data security was ensured through record-keeping protocols and use of a lockbox. The field survey teams received supervisory visits every second week during data collection.

### Statistical analysis

Paper-based questionnaires were anonymized and double-entered into an EpiData (EpiData Association, Odense, Denmark) template, and analyses were performed using the Respondent Driven Sampling Analysis Tool (RDSAT) version 5.6.0 [24]. RDSAT weighs each variable by network size of each individual. To explore the information on mobility and working patterns, descriptive statistics and comparisons were done by province. The social network size was defined as all migrants currently 15 years or older that the participant knew by first name and had seen in the past 30 days. RDSAT was used to calculate weighting of the samples to control for differences in network size and homophily of the population-based estimates for the two study districts. RDSAT software was used to estimate prevalence and confidence intervals for categorical variables other than location. All analyses were separately performed for the two study districts.

## Results

### Health messages are reaching MMPs, but information sources vary

764 participants were enrolled in Pailin and 737 in Veal Veang. Most respondents (93.7% in Pailin and 96.1% in Veal Veang) had received one or more health-related messages in the preceding 3 months. The most frequently listed health topic was malaria (89% in Pailin and 94.5% in Veal Veang), followed by HIV (39.9% in Pailin and 35.1% in Veal Veang); other main topics reported were personal hygiene, dengue fever, tuberculosis, and influenza. Primary sources of health information differed between the two districts; in Pailin, radio (57.1%, 95% CI 53.3–60.6) was the most common medium, followed by TV (31.3%, 95% CI 27.8–35.2), family, friends and neighbours (29.7%, 95% CI 25.8–33.2), and billboards (17.1%, 95% CI 14.2–20.3). In Veal Veang, the primary source was billboards (76.5%, 95% CI 73.9–80.7), followed by family, friends and neighbors (57.7%, 95% CI 54.1–62.3), and then by TV (32.4%, 95% CI 28.2–35.3) and radio (23.5%, 95% CI 20.0–26.8). Contact with health facilities or health care workers was an infrequently-listed source of information in both regions. In Pailin, health messages were most often received at home (61.8%, 95% CI 58.6–66.0) and the work place (51.8%, 95% CI 48.4–56.3); in Veal Veang it was at the village centre (58.6%, 95% CI 54.8–63.4) and while travelling (55.7%, 95% CI 51.8–59.7).

### Malaria knowledge was widespread, but some misinformation regarding means of transmission and prevention methods was found

The vast majority of respondents in both districts had heard the word ‘malaria’ at some point in their lives (98.6%, 95% CI 97.7–99.3, in Pailin and 99.6%, 95% CI 98.9–100.0, in Veal Veang). Most respondents (94.4% in Pailin and 98.2% in Veal Veang) correctly listed mosquitoes as a means of transmission. Nevertheless, an unsanitary environment (4.1% in Pailin and 24.4% in Veal Veang) and contaminated food or drink (15.2% in Pailin and 57.6% in Veal Veang) were also identified as means of transmission by a sizeable portion of respondents (Table 1). In Veal Veang, in which many migrant workers are involved in forestry, 10% of respondents associated malaria with forests. For malaria prevention, an overwhelming majority of the respondents (95.5% in Pailin and 99.1% in Veal Veang) listed mosquito nets as a way to prevent malaria, although substantial proportions also listed personal hygiene (11.4% in Pailin and 38.7% in Veal Veang) and clean surroundings (15.3% in Pailin and 43.0% in Veal Veang). Mosquito coils and protective clothing were infrequent responses in both districts (Table 1). Symptoms of malaria, including fever (83.5%, 95% CI 79.9–86.1, in Pailin, 88.7%, 95% CI 85.9–91.0, in Veal Veang), chills (87.0%, 95% CI 84.0–89.4, in Pailin, 94.9%, 95% CI 92.4–96.3, in Veal Veang), headaches (38.3%, 95% CI 34.7–42.1, in Pailin, 67.1%, 95% CI 64.5–72.2, in Veal Veang) and body aches (5.3%, 95% CI 3.6–7.2 in Pailin, 28.4%, 95% CI 25.7–32.6, in Veal Veang) were correctly identified by most respondents.

**Table 1:** Malaria knowledge: transmission and prevention

	Pailin (N = 764)	Veal Veang (N = 737)
	% (95% CI)	% (95% CI)
How is malaria transmitted?^a^		
Don’t know	4.0 (2.7–5.9)	0.8 (0.1–1.7)
Mosquitoes	94.4 (92.5–96.0)	98.2 (96.9–99.1)
Flies	0.3 (0.0–0.5)	0.8 (0.2–1.4)
Rain/weather	2.4 (–)	3.6 (2.4–5.2)
Dirty environment	4.1 (2.6–5.4)	24.4 (21.2–29.0)
Working in the sun	0.0	1.6 (0.9–2.4)
Food/drink	15.2 (12.7–17.6)	57.6 (54.0–62.1)
Spirits	1.0 (0.4–0.5)	0.4 (0.0–0.9)
Forests	3.2 (1.9–4.4)	10.0 (7.9–13.1)
Contact with sick person	0.1 (0.0–0.5)	0.5 (0.1–1.2)
How is malaria prevented?^a^		
Don’t know	1.5 (0.7–2.3)	0.6 (0.1–1.5)
Sleeping under a mosquito net	95.5 (94.2–96.8)	99.1 (98.4–99.7)
Taking preventive medicine	1.2 (0.7–1.9)	3.6 (2.1–5.0)
Using a mosquito coil	12.7 (9.7–15.5)	14.4 (11.9–17.1)
Keep house surroundings clean	15.3 (12.6–18.1)	43.0 (39.3–47.5)
Covering stagnant water	3.8 (2.6–5.0)	14.3 (12.3–17.0)
Closing house windows and doors	1.7 (0.7–2.8)	1.4 (0.6–2.4)
Personal hygiene and sanitation	11.4 (9.1–13.5)	38.7 (35.1–42.1)
Fire/smoke	1.3 (0.3–1.9)	4.7 (3.3–6.4)
Protective clothing	7.9 (6.9–11.5)	6.9 (4.4–8.4)
Mosquito spray/repellant	0.0	1.7 (0.8–2.6)

^a^More than one response was possible for these questions

### The majority of respondents slept in dwellings that were not fully enclosed, and many did not own or use LLINs or ITNs

Almost all respondents slept in makeshift structures that left them exposed to mosquitoes when not protected by a net; only 2.3% of respondents in Pailin and 0.3% in Veal Veang reported sleeping in completely enclosed dwellings. Ownership of ITNs was approximately twice as common in Veal Veang as in Pailin (53.2% vs 25.3%); if taking into account other types of treated nets, including hammock nets, net ownership stood at 54.3% in Veal Veang and 30.1% in Pailin (Table 2). A substantial proportion of respondents owned conventional treated nets instead of long-lasting insecticide-treated nets (LLINs). Respondents who did not own a net said they did not own a net because either it was unnecessary (41.1%, 95% CI 34.7–47.9, in Pailin and 23.2%, 95% CI 15.5–31.1, in Veal Veang), they did not know where to find one (26.4%, 95% CI 23.1–32.4, in Pailin and 19.5%, 95% CI 13.7–26.8, in Veal Veang), or they could not afford one (26.3%, 95% CI 19.6–31.4, in Pailin and 44.7%, 95% CI 28.8–62.7 in Veal Veang).

**Table 2:** Ownership and use of insecticide treated nets and hammock nets

	Pailin (N = 764)	Veal Veang (N = 737)
	% (95% CI)	% (95% CI)
Type of structure where the respondent slept		
Under a roof, but no walls	23.1 (20.3–25.9)	44.0 (40.1–47.6)
Incompletely enclosed	72.2 (69.0–75.2)	50.6 (46.8–54.8)
Completely enclosed	2.3 (1.4–3.4)	0.3 (0.0–0.7)
Outdoors	2.4 (1.3–3.7)	5.1 (3.3–7.4)
Own one or more		
ITNs (LLIN or non-LLIN)	25.3 (22.2–28.9)	53.2 (48.7–56.7)
LLINs	15.7 (13.7–18.3)	10.1 (8.0–12.1)
Treated hammock nets	6.2 (4.4–7.8)	3.1 (2.0–4.5)
Use of treated/untreated nets or hammocks		
ITN (treated)	57.1 (55.0–61.5)	31.6 (27.2–34.5)
ITN (unsure if treated)	0.9 (0.3–1.5)	0.0
LLIN	27.3 (23.6–29.4)	6.0 (4.3–7.9)
LLIHN	2.3 (1.2–3.5)	2.6 (1.5–3.5)
Non-long lasting treated hammock net	3.9 (2.4–5.1)	20.5 (17.6–23.7)
Untreated hammock net	0.4 (0.0–0.9)	0.0
No use of ITN/hammock net	8.1 (6.2–10.2)	39.3 (36.0–44.3)

### Bed net usage varied significantly between regions

The night before the survey, 91.9% of respondents in Pailin slept under a bed net of some type, while only 60.7% of those in Veal Veang did so. In Pailin, 57.1% slept under a conventional ITN and 27.3% slept under an LLIN, while in Veal Veang 31.6% slept under a conventional ITN and 20.5% slept under a non-long lasting treated hammock net (Table 2). Only a small proportion of conventional nets used the night before had been re-treated at some point after they were purchased (3.6%, 95% CI 2.1–6.3, in Pailin and 1.1%, 95% CI 0.0–1.7, in Veal Veang). The majority of nets had been acquired at a market or shop (55.8%, 95% CI 53.6–60.9, in Pailin and 75.9%, 95% CI 71.7–79.5 in Veal Veang), through family and friends (15.9%, 95% CI 12.7–18.7, in Pailin and 14.6%, 95% CI 10.6–17.4, in Veal Veang), as well as employers (13.7%, 95% CI 10.9–16.1, in Pailin and 6.1%, 95% CI 4.7–9.3, in Veal Veang). Most had been purchased (56.8%, 95% CI 53.1–61.0, in Pailin and 74.1%, 95% CI 69.8–78.3 in Veal Veang), but 21.9%, 95% CI 18.3–24.4, of nets in Pailin and 16.3%, 95% CI 13.0–19.9, in Veal Veang had been obtained for free; more nets had been borrowed Pailin (21.2%, 95% CI 17.8–25.6) than in Veal Veang (9.6%, 95% CI 6.5–13.1).

### More than half of participants sought private health care during their last illness

Very few respondents in either Pailin or Veal Veang had health insurance (1.7%, 95% CI 1.0–2.5, in both districts). During their last illness of any kind, less than 1% of participants had self-treated or failed to seek care. However, with respondents sometimes reporting more than one source of care, a quarter (24.6%, 95% CI 21.5–27.5, in Pailin and 25.1%, 95% CI 21.6–27.9 in Veal Veang) accessed a private clinic, a third sought care at a drug outlet during their last episode of illness (33.1%, 95% CI 28.9–37.5, in Pailin and 33.5%, 95% CI 29.1–38.1, in Veal Veang), and half went to a government health facility (49.7%, 95% CI 44.0–53.2) in Pailin and 48.9%, 95% CI 45.3–53.0, in Veal Veang).

### A significant proportion of participants were treated for malaria in the private sector

There were differences observed in the proportion treated for malaria in the previous 3 months: 13.2% (95% CI 11.1–15.8) of participants in Pailin and 30.1% (95% CI 26.4–33.8) in Veal Veang. In Pailin, 47.8% of those treated received their medication at a government health facility, 20.9% from VMWs, and one-third from private clinics or drug outlets. In Veal Veang, only 27.4% got them at a government facility, 12.6% from a VMW, and more than half from a private facility or drug outlet (Table 3). Two respondents in Pailin and one in Veal Veang were unable to access anti-malarials due to lack of availability or affordability.

**Table 3:** Treatment-seeking behaviour for malaria

	Pailin (N = 115)	Veal Veang (N = 219)
	% (95% CI)	% (95% CI)
Source of medicine		
Gov’t hospital/clinic	47.8 (38.6–57.1)	27.4 (21.4–33.5)
Private hospital/clinic	21.7 (14.1–29.4)	36.7 (30.2–43.2)
NGO	1.7 (0–4.2)	0.0
Drug outlet	11.3 (5.4–17.2)	20.0 (14.6–25.4)
Market stall/shop	0.9 (0–2.6)	4.2 (1.5–6.9)
Dispensary at work	0.9 (0–2.6)	0.5 (0–1.4)
VMW	20.9 (13.3–28.4)	12.6 (8.1–17.0)

### A significant proportion of malaria patients were treated without diagnostic testing

Of those treated for malaria in the past 3 months, the majority was treated with anti-malarials (94.9%, 95% CI 90.9–98.9, in Pailin and 96.9%, 95% CI 94.6–99.2, in Veal Veang). However, only 81.1% (95% CI 73.6–88.7) in Pailin and 86.6% (95% CI 82.1–91.1) in Veal Veang had received a diagnostic test, such as RDT or microscopy.

### Malaria treatment often failed to comply with national treatment guidelines

DHA–PPQ, the recently introduced first-line treatment during the data collection period, was used for only 29.9% of cases in Pailin and 19.9% of cases in Veal Veang. An artesunate–mefloquine combination and atovaquone–proguanil made up 28.6% of treatments in Pailin and 43.8% of treatments in Veal Veang (Table 4). There was a substantial presence of chloroquine in both sites. Three-quarters in both districts reported taking a 3-day regimen. The vast majority reported fully adhering to their treatment (97.3%, 95% CI 94.3–100, in Pailin and 94.5%, 95% CI 91.5–97.6, in Veal Veang). Among the handful that did not, side effects and symptom improvement were the predominant reasons given.

**Table 4:** Characteristics of treatment obtained

	Pailin (n = 115)	Veal Veang (n = 219)
	% (95% CI)	% (95% CI)
Type of medication		
Don’t know	16.5 (9.6–23.4)	8.7 (4.9–12.4)
Artesunate monotherapy	2.6 (0–5.6)	5.0 (2.1–7.9)
Artesunate + mefloquine	15.6 (8.9–22.4)	34.7 (28.3–41.1)
DHA + piperaquine	29.6 (21.1–38.0)	19.6 (14.3–24.9)
Atovaquone + proguanil	13.0 (6.8–19.3)	9.1 (5.3–13.0)
Mefloquine only	0.0	1.4 (0–2.9)
Quinine	4.3 (0.6–8.1)	1.8 (0.0–3.6)
Doxycycline	0.9 (0–2.6)	0.0
Chloroquine	13.9 (7.5–20.3)	16.0 (11.1–20.9)
Paracetamol	0.0	6.4 (3.1–9.7)
“Drug cocktail”	0.0	5.0 (2.1–7.9)
Other	1.7 (0–4.2)	0.5 (0–1.4)
Duration of treatment (days)		
1	2.7 (0–5.7)	1.4 (0–2.9)
2	3.5 (0.1–7.0)	5.5 (2.5–8.6)
3	75.2 (67.1–83.3)	76.6 (70.9–82.3)
7	10.6 (4.9–16.4)	5.5 (2.5–8.6)

## Discussion

The study found many differences between MMPs in Pailin and in Veal Veang, differences which can in part be explained by their different occupations and locations. Participants in Veal Veang had more misconceptions regarding malaria transmission and prevention, lower use of insecticide treated nets or hammocks, higher rate of malaria diagnosis in the previous 3 months, lower use of government facilities or VMWs for malaria treatment-seeking, and lower use of the first-line anti-malarial for those diagnosed with malaria. In Pailin, where MMPs tend to stay longer and have more stable households [16], health education was mostly provided by media at home or in the workplace, whereas in Veal Veang, where workers often live in makeshift dwellings and travel frequently [16], migrants received malaria education mostly while travelling, or at the village centre, and mostly from billboards and friends and family. An RDS study conducted among Cambodian migrants in Thailand also found that long-term migrants were far more likely to receive malaria education from television and radio than short-term migrants [24, 25]. This may explain why malaria misconceptions were sometimes three or four times more prevalent in Veal Veang than in Pailin; while media provided Pailin migrants with a steady source of detailed, accurate, government and NGO-sponsored information, workers in Veal Veang spending most of their time deep in the forest had decreased access to these sources. Billboards, the most common source of malaria education in Veal Veang, were also a source of official malaria education, but the nature of billboards limits the detail with which information can be presented, particularly compared to the television and radio spots being broadcast in Pailin. Nevertheless, further research is required to understand whether there were other barriers, such as MMP literacy, that may have limited the effectiveness of this intervention, or whether the content of the billboards could have been presented more effectively. Regardless, the above data suggests that in regions where migrant worker profiles are similar to those of Veal Veang migrants, village centres should be targeted aggressively for educational purposes. Recent publications show that interpersonal communications from VMWs, who accounted for only 5% of health messages transmitted in this study, are extremely effective and should be a primary source of malaria education [26].

It is important to consider that in the months preceding data collection for this study, a concerted effort to reduce drug-resistant malaria in Pailin was underway [27]. This undoubtedly also contributed to the fact that Pailin migrant workers consistently had better knowledge and behaviour outcomes than Veal Veang migrants, especially because the data regarding health-seeking behaviours for non-malaria illnesses was virtually identical for both districts.

### Potential effect on drug resistance

The study found that a large proportion of the health services accessed by study participants had been provided by the private sector. In a context of MDR malaria, this information is alarming. It has been widely documented that private healthcare in Cambodia is a significant driver of parasite resistance, and negatively impacts individual patients’ prognoses, because the private sector does not adhere to national treatment guidelines and often provides substandard or counterfeit drugs [28–30]. Approximately 10% of participants who had been recently treated for malaria did not have a confirmed diagnosis from a positive diagnostic test, which is also concerning Nevertheless, the 2010 Containment Survey found both of these issues were present among the general population as well, with very similar rates [31].

One of the most significant findings of this study was the use of anti-malarials in contravention of the National Treatment Guidelines. There was a substantial presence of chloroquine (13.9% in Pailin and 16.0% in Veal Veang) and other anti-malarial regimens with documented resistance. DHA–PPQ, the first-line treatment according to national guidelines at the time, was only used in 30% of cases in Pailin and 20% of cases in Veal Veang.

The first-line treatment rates are surprising, particularly in Pailin, where DHA–PPQ became the first-line treatment more than 2 years before it did so in the rest of the country. However, once again the data is consistent with that of the general population; the 2010 Containment Survey found that artesunate–mefloquine was given to 79.7% of participants who had reported that they had a malaria-type of fever [31]. Consistently unreliable supply chains have been identified as a significant barrier to malaria treatment in Cambodia [26], but more research is needed in order to understand all the factors that led to such poor implementation here. This is particularly crucial now that Cambodia has once again changed its first-line treatment. A failure to expediently discontinue use of DHA–PPQ now that there is documented clinical failure will facilitate the spread of drug resistant parasites, and could have a tragic impact on global malaria morbidity and mortality rates [32, 33].

Overall, the presence of drug resistance drivers among MMPs identified in this study are concerning, particularly because the mobility of MMPs is a major factor in the spread of drug-resistant malaria [9]. Similar findings among the general population suggest that the problem is a national one and not MMP-specific, however, interventions that can resolve these issues in the general population may not be effective among MMPs; they should be targeted with tailored interventions that take into account their heterogeneity and specific socio-geographical contexts.

### Use of LLINs and ITNs

The rate of net usage in Pailin was a remarkable (91%) higher even than that of the general population (83%) according to the 2010 Containment Survey [32], and may in part be a reflection of behaviour change communication interventions that were ongoing at the time in Pailin [34]. The high use despite low ownership can be explained by the fact that migrant workers in Pailin tend to live communally, which includes sharing a bed, and consequently a bed net, among several individuals [16]. At the same time, the proportion of those who said they had slept under a borrowed net was more than double that in Veal Veang; it is likely that a lending scheme that had been recently piloted in Pailin had a positive impact on migrant workers’ access to nets [35]. A large-scale lending scheme should be made available to all MMPs with characteristics similar to those of Pailin, as a single net can go a long way by providing coverage to several individuals.

Despite a much higher rate of ownership in Veal Veang than in Pailin, net usage the night before the survey was far lower, and was 20% lower than among the general population as reported by the 2010 Containment survey [25]. More research is needed to determine the drivers of low net use in Veal Veang in particular, and forest or periodic workers in general, especially since the intensified interventions in Pailin make it difficult to compare between the two MMP groups. It is, however, interesting to note that Cambodian migrant workers in Thailand were found to have a net usage rate of 97% in a prior RDS study [23, 24]. Lessons learned from interventions in Thailand that led to this success can perhaps be applied among forest workers in Cambodia. Innovative educational and other interventions tailored to their unique context should be developed and implemented. This is of priority, as forest workers in Cambodia are at most risk of contracting malaria.

## Study limitations

There are a number of limitations to this study. The data obtained was entirely self-reported, and subject to recall and desirability biases. As all participants received an ITN, this may have artificially elevated the number that said they knew about the use of ITNs as a preventive measure, or the reported use. Additionally, RDS methodology has its own inherent weaknesses, including purposive site selection with a primary concern of selecting sites convenient for the target population and purposive selection of the original seeds who recruit other participants, subject to those who are known to local health authorities. RDS is used to recruit among peer groups, which can lead to selection bias; it could also bias results, as peer networks tend to influence each other. It is also likely that anyone working in conditions in which the employer did not allow for travel outside of the premises did not participate, so one of the most vulnerable sectors of the target population is not accounted for.

## Conclusion

Understanding MMP heterogeneity will be necessary in order to eliminate malaria in Cambodia; targeting them is a priority, as their high mobility and low access to adequate prevention and treatment is one of the major contributors to the spread of drug resistant parasites across three cross-border points in four countries in the Greater Mekong Sub-region [8, 11]. Without an understanding of their unique contexts, practices and behaviours, this cannot be done effectively. Lessons learned and documented in this study have been applied to the design of efforts to reach MMP, and observations regarding low use of first line treatment are applicable as Cambodia is once again several months into the process of changing its first line treatment for malaria.

## Abbreviations

ACT: artemisinin-based combination therapy
CHW: community health worker
HC: health centre
HCW: health care workers
ITN: insecticide-treated net
LLIN: long-lasting insecticide-treated net
LLIHN: long-lasting insecticide-treated hammock net
MMP: mobile and migrant population
RDS: respondent-driven sampling
RDT: rapid diagnostic test
VMW: village malaria worker
WHO: World Health Organization
VHV: village health volunteer
